# The Attenuating Effect of the Intraovarian Bone Morphogenetic Protein 4 on Age-Related Endoplasmic Reticulum Stress in Chicken Follicular Cells

**DOI:** 10.1155/2020/4175613

**Published:** 2020-06-08

**Authors:** Jinwei Yao, Yanfen Ma, Xin Lin, Shuo Zhou, Yuling Mi, Caiqiao Zhang

**Affiliations:** College of Animal Sciences, Zhejiang University, Hangzhou 310058, China

## Abstract

In the poultry, only less than 5% primordial follicles in the ovary can develop into the prehierarchical follicles (PHFs) leading to progressive development, ovulation, and egg formation. This low rate of recruitment indicates a huge potential for improvement of the laying performance. A great reduction in egg production is caused by aging with extensive follicular atresia. In this study, age-related changes in the laying performance and ovarian status were compared between the peak-lay (D280) and aged (D580) chickens. Subsequently, a cross coculture of PHFs and granulosa cells (GCs) from D280 or D580 hens was adopted to reveal the mechanism of declined follicle development. Results showed that persistent endoplasmic reticulum (ER) stress in GCs of the aged hens was accompanied with intensified apoptosis. Bone morphogenetic protein 4 (BMP4) secreted by GCs of PHFs in D280 hens was capable of relieving ER stress and improving follicular dominance for selection in D580 hens. During this action, BMP4 reduced free calreticulin (CALR, an ER marker) content and attenuated cell apoptosis in PHFs of D580 hens via the PERK-CHOP-BCL2/caspase3 or CALR-Ca^2+^-BCL2-caspase12 pathway. Furthermore, BMP4 prevented follicular atresia by promoting production of steroid hormones to improve survival of GCs in PHFs from the aged hens. In conclusion, intensified ER stress and apoptosis occurred in GCs of PHFs in aged chickens, while BMP4 secreted by GCs was capable of improving follicular viability by alleviating ER stress to promote follicular development.

## 1. Introduction

In the chicken, the rate of egg production decreased markedly around 580 days of age, which seriously reduces the commercial value of egg production. In order to improve the egg yield by extending the laying period, it is necessary to explore the deteriorating mechanisms of the laying performance that are caused by aging. The rate of egg production is related to the function of ovaries and quality of follicles in laying hens [1, 2]. Previous studies reported that the level of intrafollicular follicle-stimulating hormone receptor (FSHR) was correlated with the predominance of prehierarchical follicles (PHFs) [3, 4]. However, few studies have reported the molecular mechanism involved in the decrease of follicular dominance in the aging hens. Insulin-like growth factor I secreted by granulosa cells (GCs), as well as bone morphogenetic protein (BMP) and estrogen, is reported to play crucial roles in the growth and development of follicles [5–7]. GCs are destined to undergo apoptosis when these factors are depleted. Moreover, in the early atretic follicles, GCs are the first cell type to undergo apoptosis [8]. Therefore, formulation of the relationship between cytokines and GC apoptosis is extremely important for improving follicular predominance and subsequent efficient follicular development.

Endoplasmic reticulum (ER) is an important organelle in which many cellular reactions occur, especially synthesis and folding of proteins as well as the storage and release of Ca^2+^ [9]. The unfolded protein response is induced by the accumulation of misfolded proteins. Furthermore, ER stress is induced when the unfolded proteins accumulated or the misfolded proteins are not cleaned up promptly. Imbalance of Ca^2+^ concentration in the ER can also induce ER stress [10]. As markers of ER stress, calreticulin (CALR) and glucose-regulated protein 78 (GRP78) were upregulated when ER stress occurs [11, 12]. CALR is the major calcium-binding molecular chaperone in ER and is responsible for controlling Ca^2+^ release into cytoplasm to participate in the reactions of the misfolded proteins and regulate cell apoptosis [13]. Three ER transmembrane mediators (inositol requiring (IRE), protein-like endoplasmic reticulum kinase (PERK), and activating transcription factor (ATF4)) are dissociated with GRP78 and activated when ER stress occurs. After dissociation with GRP78, PERK is phosphorylated to p-PERK, then further phosphorylates the downstream target protein eIF2 alpha site leading to ATF4 activation. The persistence of ATF4 activation enables CHOP to translocate from cytoplasm to nucleus, subsequently initiating the apoptosis process [14, 15]. Moreover, increased CALR was transferred from ER to cell membrane for triggering the PERK/CHOP pathway, which in turn upregulated CHOP expression as well as inhibited BCL2 expression, and finally resulted in cell apoptosis through the caspase3 pathway [16].

Although there are many studies on the apoptosis induced by persistent ER stress, it is unclear whether apoptosis in ovarian follicles of the aging hens is associated with ER stress. BMP4 is reported to play a positive role in poultry follicular development as this cytokine promotes differentiation of GCs in the PHFs, boosts expression of FSHR in GCs, and accelerates development of PHFs into hierarchical follicles [17]. In addition, BMP4 promoted survival of GCs and increased the diameter of primordial follicles and secondary follicular oocytes [18]. These functions of BMP4 are mainly achieved by upregulating the expression of several steroidogenic enzymes (including *CYP11a1*, *HSD3B2*, *CYP17a1*, and *CYP19a1*) to inhibit androgen synthesis in follicular theca cells (TCs) and promote estrogen production in mouse GCs [19]. On the other hand, prolactin secreted by the anterior pituitary gland could induce the nesting behavior of the poultry, thereby reducing egg production and reproduction performance. Increased expression of prolactin receptor (PRLR) was involved in this process [20]. Elevated expression of PRLR led to an increased nesting rate of chickens, accompanied with degeneration of the fallopian tubes and ovaries, finally resulting in ceasing of egg laying [21]. Since BMP was reported as a PRLR inhibitory factor, it is suggested that BMP may regulate ovarian function through interaction with the PRLR signaling pathway, thereby increasing the poultry production rate [22]. In addition, a previous study revealed an antagonistic relationship between BMP and CALR [23]. Therefore, we assume that BMP4 may promote survival of GCs by relieving the ER stress of GCs in the aging hens.

Based on the pivotal roles of BMP in follicular development, this study was designed to compare the morphological and biochemical changes between the peak-lay and aging hens. In addition, RNA-sequence (RNA-seq) analysis was used to manifest the differentially expressed genes. Subsequently, a cross coculture of ovarian follicles and/or GCs from young and aging laying chickens was adopted to elucidate functions of intraovarian cytokines in preventing age-related ER stress involved in the decreased laying performance of the aging hens.

## 2. Materials and Methods

### 2.1. Animals

The yellow-feathered peak-lay hens (D280) and aged hens (D580) were purchased from Ningbo Zhenning Animal Husbandry Co. Before the formal experiment, the hens were accustomed to the campus animal house with free access to water and feed for two weeks until the egg production rate reached stability. The diet compositions are listed in Table S1. All experimental procedures were performed in accordance with the Guidelines for Care and Use of Laboratory Animals of Zhejiang University with the approval reference number ZJU20170660.

### 2.2. Tissue Collection

Three hens of two ages were randomly selected for sampling each time. The hens were scarified by bleeding after anesthesia with pentobarbital sodium. The abdominal cavity was opened, and the functional ovaries were carefully removed. The entire ovary was immersed in phosphate-buffered saline (PBS) to remove blood cells as clean as possible. Then, the PHFs were collected for morphological observation, analysis of Western blot, or quantitative real-time polymerase chain reaction (qRT-PCR).

### 2.3. Culture of SWFs

Intact small white follicles (SWFs, 2-4 mm) were cultured in Dulbecco's Modified Eagle's Medium (DMEM high glucose, Hyclone, Tauranga, New Zealand) in 24-well plates, with 2 SWFs in each well. Each well contained 500 *μ*L DMEM supplemented with 5% fetal calf serum (FCS, Hyclone, Tauranga, New Zealand), 1×ITS (10 *μ*g/mL insulin, 5 *μ*g/mL transferrin, and 30 nM selenite, Sigma-Aldrich), 100 IU/mL penicillin, and 100 *μ*g/mL streptomycin. All cultures were transferred into an incubator at 38.5°C with 5% CO_2_ for 72 h. The media were renewed every 24 h, and 10 *μ*g/mL bromodeoxyuridine (BrdU, Sigma-Aldrich, St. Louis, MO) was added to the medium at the last 24 h. After culture, SWFs were fixed for morphological observation, Western blot, or qRT-PCR.

### 2.4. Isolation and Culture of GCs from SYFs

For the separation of GCs, the small yellow follicles (SYFs, 6-8 mm) were torn apart with tweezers, and the yolk was extruded. After washing follicles with ice-cold PBS three times, the granulosa layers (GLs) were carefully scraped away from the inner wall of the follicles. The collected GLs were washed several times in PBS until the yolk was removed and then were digested with 1 mg/mL collagenase II (Gibco, Grand Island, NY) for 3 min at 37°C. The dispersed GCs were filtered through a 74 *μ*m mesh. The filtrate was centrifuged at 1000 rpm for 4 min, and the precipitate was washed three times with ice-cold DMEM. The cells were seeded at a density of 1 × 10^5^ cells/well on collagen-coated 24-well plates with 500 *μ*L DMEM supplemented with 1×ITS, 5% FCS, 100 IU/mL penicillin, and 100 *μ*g/mL streptomycin and incubated at 38.5°C and 5% CO_2_.

### 2.5. Coculture of SWFs and GCs

GCs from the SYFs were seeded at a density of 1 × 10^5^ cells/well and placed on a 0.4 *μ*m insert hanging chamber (Merck Millipore, Billerica, MA) in a 24-well plate, with two SWFs in each well [24]. Each hanging chamber and 24-well plate contained 500 *μ*L complete DMEM that was supplemented with 5% FCS, 1×ITS, 100 IU/mL penicillin, and 100 ng/mL streptomycin. Coculture conditions were identical with the methods for the culture of SWFs and GCs.

### 2.6. Treatment of the Cultured SWFs and GCs

For screening the suitable dosage of BMP4, the SWFs were treated with BMP4 at 0, 10, 100, or 1000 ng/mL (MCE, HY-P7007, Shanghai, China). Based on morphological changes, cell apoptosis, and cell proliferation, BMP4 concentration of 100 ng/mL was selected as the optimal concentration for further studies. Next, in order to compare the effects of BMP4 and GCs on SWFs, we set the following six groups: (1) the control group of SWFs, (2) SWFs cocultured with GCs, (3) SWFs treated with 100 ng/mL BMP4, (4) SWFs cocultured with GCs and treated with 30 nM DM-3189, a BMP receptor (BMPR) antagonist (MCE, HY-12071, Shanghai, China), (5) SWFs treated with 100 ng/mL BMP4 and 30 nM DM-3189, and (6) SWFs treated with 30 nM DM-3189. All of the cultures were maintained in a humidified atmosphere with 5% CO_2_ at 38.5°C.

### 2.7. Morphological Observation

All tissues and cells were washed more than three times in cold PBS and immersed in 4% paraformaldehyde and fixed at 4°C over 24 h. After fixation, tissues and cells were rinsed by running water, and the cells were used for subsequent immunohistochemistry. The tissues were dehydrated by graded ethanol and immersed in 60°C paraffin for more than 4 h and embedded. The paraffin section was prepared at 4 *μ*m for immunohistochemistry (IHC), BrdU incorporation, and TUNEL assay. Hematoxylin and eosin (HE) staining was carried out according to a conventional protocol. IHC and immunofluorescence (IF) staining were referred to a previous method [1]. The antibodies used for IHC and IF were as follows: mouse anti-GRP78 (1 : 50, sc-376768), CHOP (1 : 50, sc-46661, Santa Cruz Biotechnology, Santa Cruz, USA), rabbit anti-FSHR (1 : 100, A1480, ABclonal Technology, Wuhan, China), anti-ATF4 (1 : 100, ET1612-37), anti-caspase3 (1 : 100, ET1602-39), anti-BMPR1A (1 : 100, R1510-1), anti-CYP19a1 (1 : 100, ER1802-38), mouse anti-calreticulin (1 : 100, EM1701-61, HuaBio, Hangzhou, China), rabbit anti-STAR (1 : 100, CSB-PA022798LA01HU), anti-CYP11a1 (1 : 100, CSB-PA006389LA01HU, Cusabio, Wuhan, China), or mouse anti-BrdU antibody (1 : 200, AB_2314035, G3G4, DSHB, IA, Iowa, USA). Positive cells were stained using DAB, and the images were captured using an Eclipse 80i microscope (Nikon, Tokyo, Japan). IX70 fluorescence microscopy (Olympus, Tokyo, Japan) was used to visualize the fluorescent images of the cells and tissues on the slides.

### 2.8. TUNEL Assay

Cell apoptosis was detected using a TUNEL BrightGreen Apoptosis Detection Kit (Vazyme, Nanjing, China) according to the kit instruction.

### 2.9. Western Blot

The SWFs were fully lysed by an ice-cold RIPA solution, and the SWF-lysate was centrifuged at 12000 rpm for 20 min at 4°C. Protein concentration in the supernatants was measured by a BCA protein assay kit (Nanjing Jiancheng Bioengineering Institute, Nanjing, China). Samples of 20 *μ*g of protein were applied to SDS-PAGE glue, and the protein was transferred to a methanol-activated polyvinylidene difluoride (PVDF) membrane (Millipore, Bedford, MA, USA) after running for 30 min at 80 V and 90 min at 120 V. After blocking PVDF with 5% skim milk, the immunoassay was detected with the corresponding primary antibodies and then incubated with the secondary antibodies. The bands were visualized by an enhanced chemiluminescence (ECL) kit (Bio-Rad, Hercules, USA). A ChemiScope 3400 Mini machine (Clinx, Shanghai, China) was used to detect the signal intensity. The antibodies used for Western blot were as follows: mouse anti-GRP78 (1 : 1000, sc-376768), CHOP (1 : 1000, sc-46661, Santa Cruz Biotechnology, Santa Cruz, USA), rabbit anti-caspase12 (1 : 1000, NBP1-76801), PERK (1 : 1000, NBP1-80930, Novus, USA), phospho-PERK (1 : 1000, DF7576, Affinity Biosciences, OH, USA), anti-ATF4 (1 : 1000, ET1612-37), anti-caspase3 (1 : 100, ET1602-39), and mouse anti-calreticulin (1 : 100, EM1701-61, HuaBio, Hangzhou, China).

### 2.10. RNA Extraction, qRT-PCR, and RNA-seq Analysis

A TRIzol reagent (Invitrogen, Carlsbad, CA) was used to extract total RNA from follicles. The cDNA was generated from 2 *μ*g total RNA using a HiScript II 1st Strand cDNA Synthesis Kit (Vazyme, Nanjing, China), following the manufacturer's protocol. The qRT-PCR was performed using a SYBR® Premix Ex Taq™ Kit (Takara, DRR420A, Kyoto, Japan) on an ABI 7500HT Real-Time PCR Detection System (Applied Biosystems, Foster City, USA), with the following conditions: 95°C for 10 min and then 40 cycles of 95°C for 30 s, 64°C for 34 s, and 72°C for 30 s. Comparisons of expression levels were determined by the 2^−*ΔΔ*Ct^ formula method normalized to *β*-actin. The sequences for primers are listed in Table 1. The RNA-seq was carried out according to a previous study [25].

### 2.11. Measurement of BMP4 Level

In order to measure the level of BMP4, the medium in the coculture system was collected and the concentration of BMP4 was detected by an Elabscience Human BMP4 ELISA Kit (E-EL-H0012c, Wuhan, China) according to the instruction.

### 2.12. Transmission Electron Microscopy (TEM)

The TEM was referred to a previous study [26]. Tissues were soaked in 2.5% glutaraldehyde over 24 h. The fixed intact follicles were dehydrated in gradient ethanol and fixed in 1% osmium tetroxide buffer for 1.5 h, then dehydrated in acetone, and embedded in LX-112 epoxy resin. The blocks were sectioned using a Leica EM UC7 ultramicrotome (Leica Microsystems GmbH, Wetzlar, Germany), and the ultrathin sections were mounted on copper-coated grids. The ultrathin sections were stained with uranyl acetate and alkaline lead citrate for 5 to 10 min. Finally, the cell ultrastructure was observed using a transmission electron microscope (Tecnai G2 Spirit 120 kV FEI Company, Hillsboro, USA).

### 2.13. Statistical Analysis

All experiments were repeated three times. Data were expressed as the mean ± standard error of the means and analyzed by the one-way ANOVA and independent sample *t*-test and Tukey's multirange test using the SPSS 20.0 software (SPSS Inc., Chicago, USA). *p* < 0.05 was considered to be a statistically significant difference.

## 3. Results

### 3.1. Comparison of Follicle Profiles and Histological Structure of D280 vs. D580 Hens

**Figure 1:**
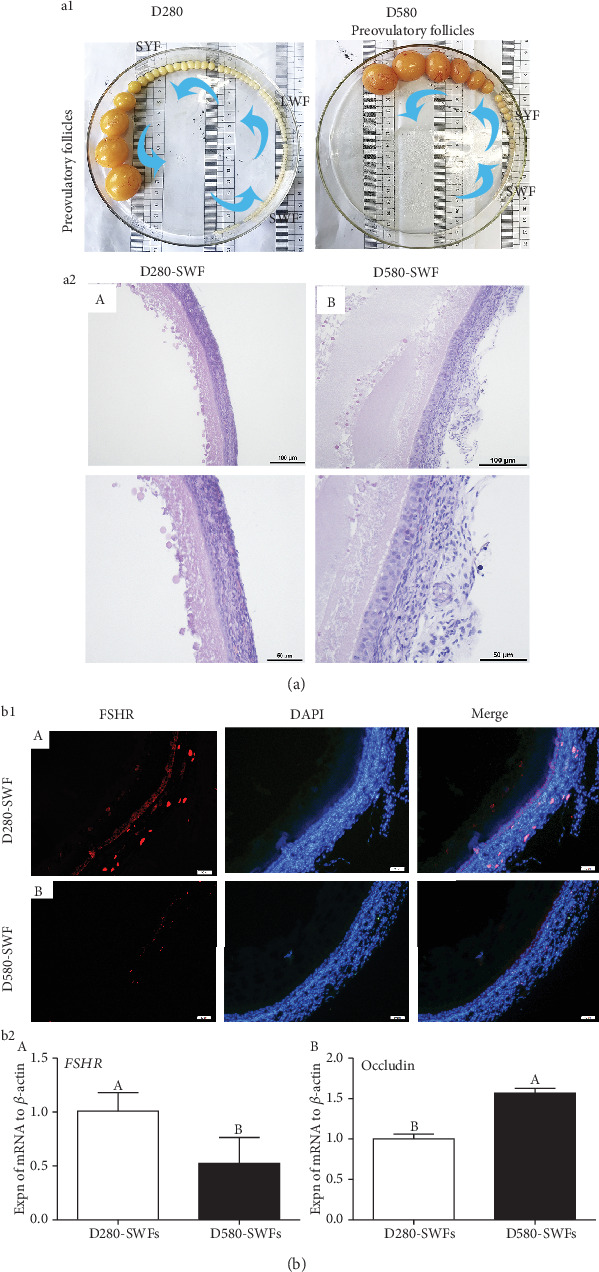
Follicular morphology ((a1), (a2)) and expression of *FSHR* and occludin ((b1), (b2)) in SWFs of the D280 and D580 hens. HE was used to evaluate the morphology of SWFs. Scale bars: (a2) 100 *μ*m (20x) and 50 *μ*m (40x); (b1) 50 *μ*m. Analysis by qRT-PCR showed that *FSHR* mRNA ((b1), (b2) A) decreased in D580-SWFs while occludin mRNA ((b2) B) increased. Values were the mean ± SEM of three experiments. Different lowercase letters indicated significant difference (*p* < 0.05).

In order to explore the differences during the aging process in the functional left ovaries of hens, we isolated all visible follicles protruding from the surface of ovaries on D280 and D580. Results showed that the number of PHFs in ovaries fell from about 60 to 40 in D280 as compared to D580 hens (Figure 1(a, a1)). HE staining showed that the overall follicular structure displayed similar architecture without significant differences between the D580 and D280 hens as follicles from both ages manifested integral structure with clear boundary between GL and TL and closely arranged GCs (Figure 1(a, a2)).

### 3.2. Decreased Function in PHFs of Aging Hens

Results of IF staining showed that expression of FSHR in the GL of PHFs from D580 hens was lower than that from D280 hens (Figure 1(b, b1)). Furthermore, the qRT-PCR result showed that expression of *FSHR* mRNA was decreased while occludin mRNA was increased in SWFs of D580 hens compared with D280 hens (Figure 1(b, b2)).

### 3.3. Increased ER Stress in GCs of SWFs from D580 Hens

**Figure 2:**
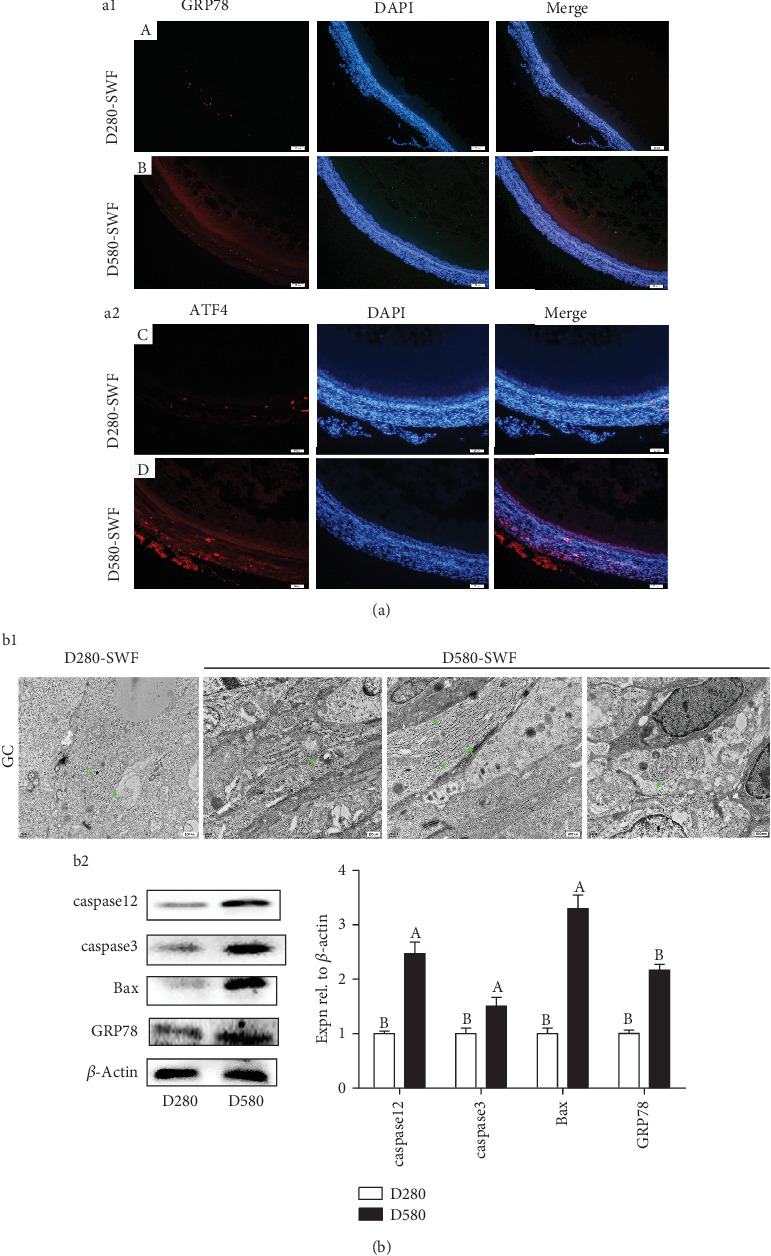
Age-related ER stress in SWFs of the D280 and D580 hens. IF staining was used to observe the localization of GRP78 ((a1) A and B) and ATF4 ((a2) C and D) in SWFs of hens, and red fluorescence was used to mark GRP78 and ATF4. Scale bar: 50 *μ*m. TEM was used to observe the ER morphology (b1) of the SWFs, and green arrows pointed to the ER. Western blot and grey analysis of caspase3, caspase12, Bax, and GRP78 (b2) expression in SWFs. Values were the mean ± SEM of three experiments. Different lowercase letters indicated significant difference (*p* < 0.05).

In order to investigate the ultrastructure changes in follicles, morphology of different follicular cells was detected with TEM. Results showed that the rough ER was uniform in size with normal morphology in D280-SYF-GCs while vacuolization, hyperplasia, and concentric circles appeared in D580-SYF-GCs (Figure 2(b, b1)). Therefore, it was inferred that the ER in GCs of PHFs of aging hens may undergo degenerative changes. Western blot analysis showed that levels of relevant proteins (caspase12, caspase3, GRP78, and Bax) were elevated in D580-SWFs compared with D280-SWFs (Figure 2(b, b2)). Moreover, the IF result revealed that GRP78 and ATF4 were highly expressed in the GCs of D580-SWFs (Figure 2(a)).

### 3.4. Effect of Aged GCs on ER Stress in PHFs of D280 Hens

**Figure 3:**
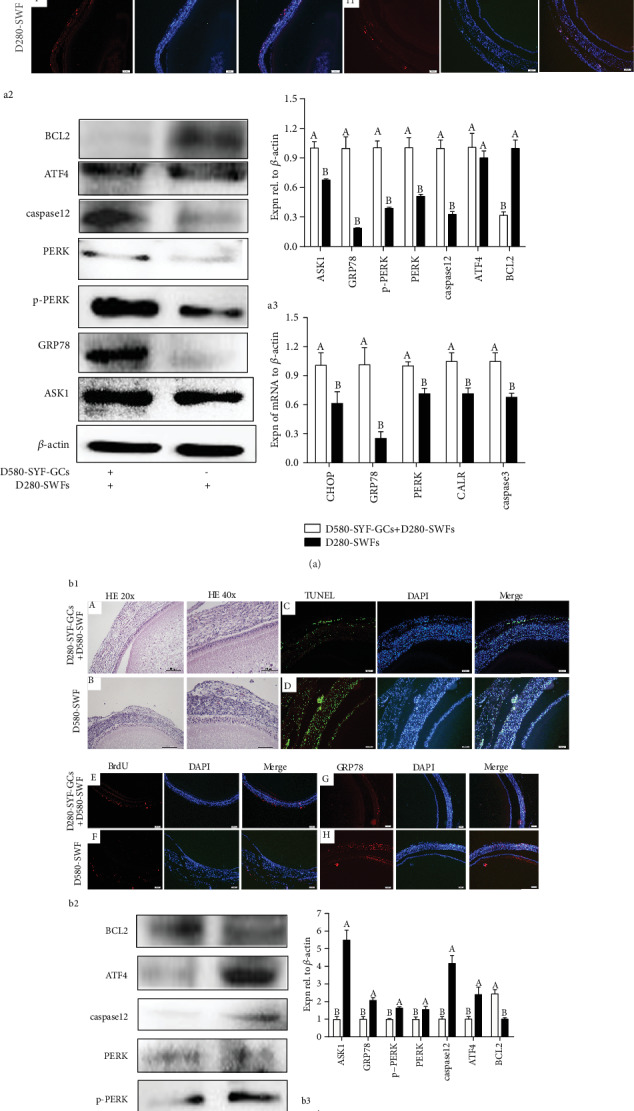
GCs dominated follicular ER stress in chicken PHFs. Morphology of follicles ((a1) A and B, (b1) A and B) after 72 h culture. Changes in the TUNEL index in SWFs ((a1) C and D, (b1) C and D) after coculture with GCs. Green: apoptotic cells. BrdU was used to label the cells ((a1) E and F, (b1) E and F) that were proliferating (red). After coculture of the D580-SYF-GCs with D280-SWFs, ER stress marker GRP78 ((a1) G and H) was increased in the GCs. After coculture of the D580-SWFs with D280-SYF-GCs, the ER marker GRP78 ((b1) G and H) was reduced in both GCs and TCs. Scale bar: 20 *μ*m. Western blot and grey analysis of BCL2, p-PERK, ATF4, caspase12, ASK1, and GRP78 (a2) expression in D280-SWFs with D580-SYF-GCs in coculture. Western blot and grey analysis of BCL2, p-PERK, ATF4, caspase12, ASK1, and GRP78 (b2) expression in D580-SWFs with D280-SYF-GCs in coculture. By qRT-PCR analysis, *CALR*, *PERK*, *GRP78*, and *CHOP* mRNAs (a3) increased in D280-SWFs (coculture with D580-SYF-GCs). The mRNAs of *CALR*, *PERK*, *GRP78*, and *CHOP* (b3) decreased in D580-SWFs (coculture with D280-SYF-GCs). Values were the mean ± SEM of three experiments. Different lowercase letters indicated significant difference (*p* < 0.05).

Microporous insert membranes were adopted for the coculture system. Though separation of the TL and GL appeared after 72 h culture, there were no significant differences in D280-SWFs in spite of the presence of the cocultured D580-SYF-GCs (Figure 3(a, a1) A and B). Furthermore, the TUNEL assay showed that few apoptotic cells were distributed in the TL of D280-SWFs, but there were increased apoptotic cells in both GL and TL of the D280-SWFs after coculture with D580-SYF-GCs for 72 h (Figure 3(a, a1) C and D). Meanwhile, results from BrdU incorporation showed that few positive cells were presented in the TL of D280-SWFs after 72 h coculture with D580-SYF-GCs (Figure 3(a, a1) E and F). Moreover, the IF result showed that an ER stress marker of GRP78, as well as TUNEL-labeling cells and BrdU-positive cells, was colocalized within follicles, and the expression level of GRP78 in the GCs of D280-SWFs was increased after coculture with D580-SYF-GCs (Figure 3(a, a1) G and H). Consistent with the IF result, Western blot experiment further revealed that a significant increase in ATF4, caspase12, PERK, p-PERK, GRP78, and ASK1 expression occurred in D280-SWFs by D580-SYF-GC coculture while the anti-apoptosis-related protein BCL2 was decreased (Figure 3(a, a2)). The expression pattern of ER stress-related mRNAs (*CHOP*, *GRP78*, *PERK*, *CALR*, and caspase3) was similar to the changes that were achieved with the IF and Western blot experiments (Figure 3(a, a3)).

### 3.5. Effect of Active GCs on ER Stress in PHFs of D580 Hens

By HE staining, we found no significant difference in ovarian follicles between the D280 and D580 hens (Figure 3(b, b1) A and B). On the contrary, the number of apoptotic cells in D580-SWFs cocultured with D280-SYF-GCs was decreased significantly as compared with D580-SWFs, especially in the GL (Figure 3(b, b1) C and D). In addition, the BrdU incorporation result showed that percentages of the proliferating cells in GL of the D580-SWFs were increased significantly by D280-SYF-GC coculture (Figure 3(b, b1) E and F). Furthermore, IF staining showed that the expression of GRP78 in the GL and TL of the D580-SWFs was decreased by D280-SYF-GC coculture (Figure 3(b, b1) G and H). Consistent with the IF result, Western blot experiment further revealed that there was a significant decrease in ATF4, caspase12, PERK, p-PERK, GRP78, and ASK1 expression that occurred in D580-SWFs by D280-SYF-GC coculture, while the BCL2 expression was increased (Figure 3(b, b2)). The expression pattern of ER stress-related mRNAs (*CHOP*, *GRP78*, *PERK*, *CALR*, and caspase3) was similar to the IF and Western blot experiments (Figure 3(b, b3)).

### 3.6. Differentially Expressed Genes (DEGs) between the D280 and D580 PHFs

To screen the predominant factors that are responsible for follicular deterioration during aging, RNA-seq is utilized to analyze the differential transcript profiles of the D280 and D580 PHFs. Among all the DEGs, we found that the expression patterns of the BMP family and the cell proliferation-related genes were significantly different between the D280 and D580 PHFs. The expression of *BMPR*, *STAR*, *TGFB3*, *TGBR3*, *CYP11a1*, *CYP17a1*, *CYP19a1*, *CDK2*, and *PCNA* mRNAs in D580-SWFs was significantly decreased compared with D280-SWFs, while the expression of *CALR3* mRNA was remarkably increased (Table 2).

### 3.7. Effect of BMP4 on Follicular Development

**Figure 4:**
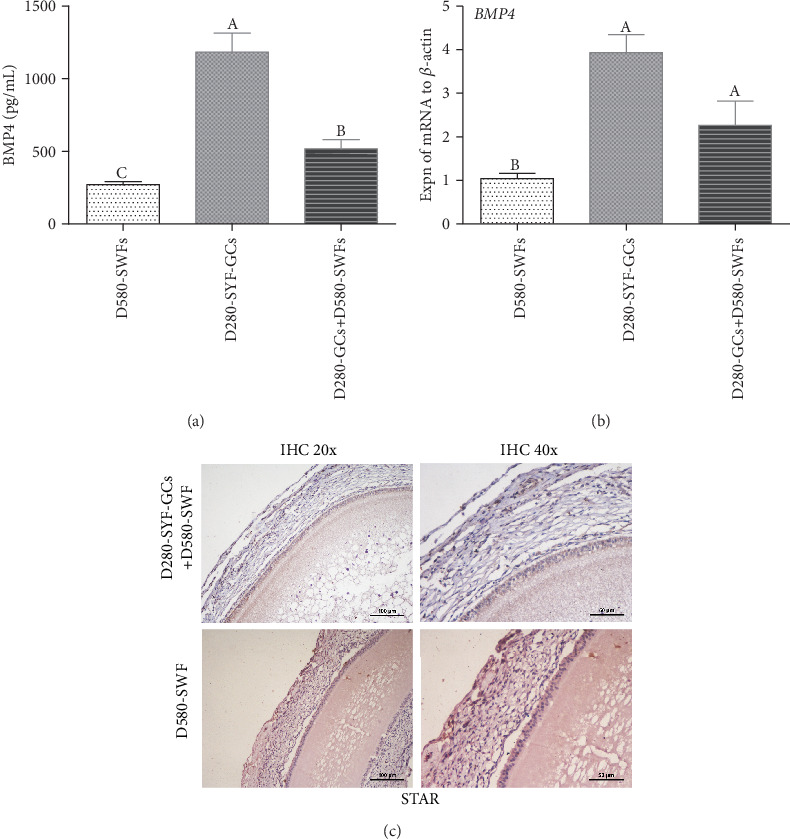
Expression of BMP4 and STAR in chicken PHFs. (a) Secretion of BMP4 determined by ELISA. (b) *BMP4* mRNA expression determined by qRT-PCR. (c) STAR immunohistochemical staining of D580-SWFs after coculture with D280-SYF-GCs for 72 h. Scale bar: 100 *μ*m (c, left) and 50 *μ*m (c, right). Values were the mean ± SEM of three experiments. Different lowercase letters indicated significant difference (*p* < 0.05).

**Figure 5:**
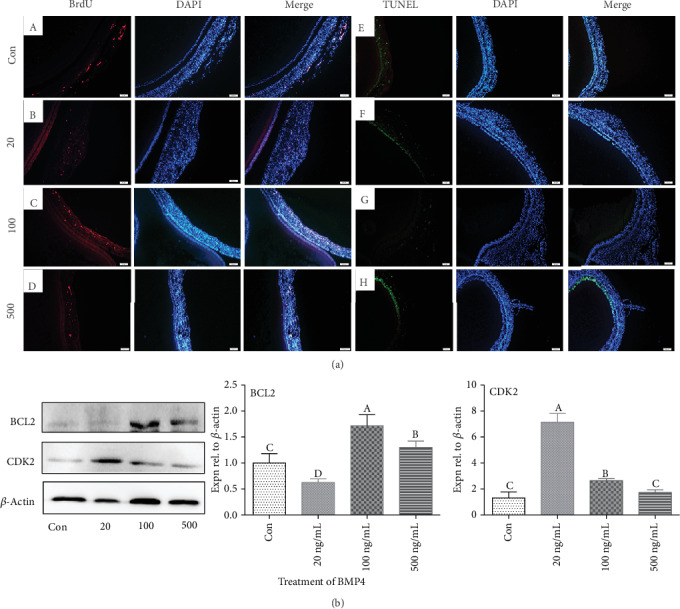
Determination of the optimal concentration of BMP4 for treatment in culture. The SWFs were incubated with BrdU. Red fluorescence represents BrdU-labeling cells ((a) A–D). Blue fluorescence represents the DAPI staining. Changes in the TUNEL index ((a) E–H) in D580-SWFs after treatment with BMP4 (20-500 ng/mL). Scale bar: 50 *μ*m. Western blot and grey analysis (b) of CDK2 and BCL2. Values were the mean ± SEM of three experiments. Different lowercase letters indicated significant difference (*p* < 0.05).

By determination of BMP4 levels in media using ELISA, we found that BMP4 in the medium of D280-SYF-GCs was higher than that of D280-SYF-GCs in coculture with D580-SWFs (Figure 4(a)). The result of qRT-PCR showed that the expression of *BMP4* mRNA was higher in D280-SYF-GCs than in D280-SYF-GCs in coculture with D580-SWFs (Figure 4(b)). IHC staining was used to detect the localization of the steroid acute regulatory protein (STAR), and results showed that STAR expression in the GL of D580-SWFs was increased after coculture with D280-SYF-GCs (Figure 4(c)). The BrdU incorporation and TUNEL assay revealed that treatment with 100 ng/mL BMP4 enhanced cell proliferation and inhibited apoptosis significantly (Figure 5(a)). Meanwhile, treatment with BMP4 at 20 ng/mL led to increased expression of CDK2 protein, while the highest expression of the antiapoptotic protein BCL2 was achieved after BMP4 treatment at 100 ng/mL (Figure 5(b)). Based on these results, 100 ng/mL of BMP4 was adopted as the optimal concentration in the next experiments.

### 3.8. Relieving Effect of BMP4 on Age-Related ER Stress and Production of Steroid Hormones

**Figure 6:**
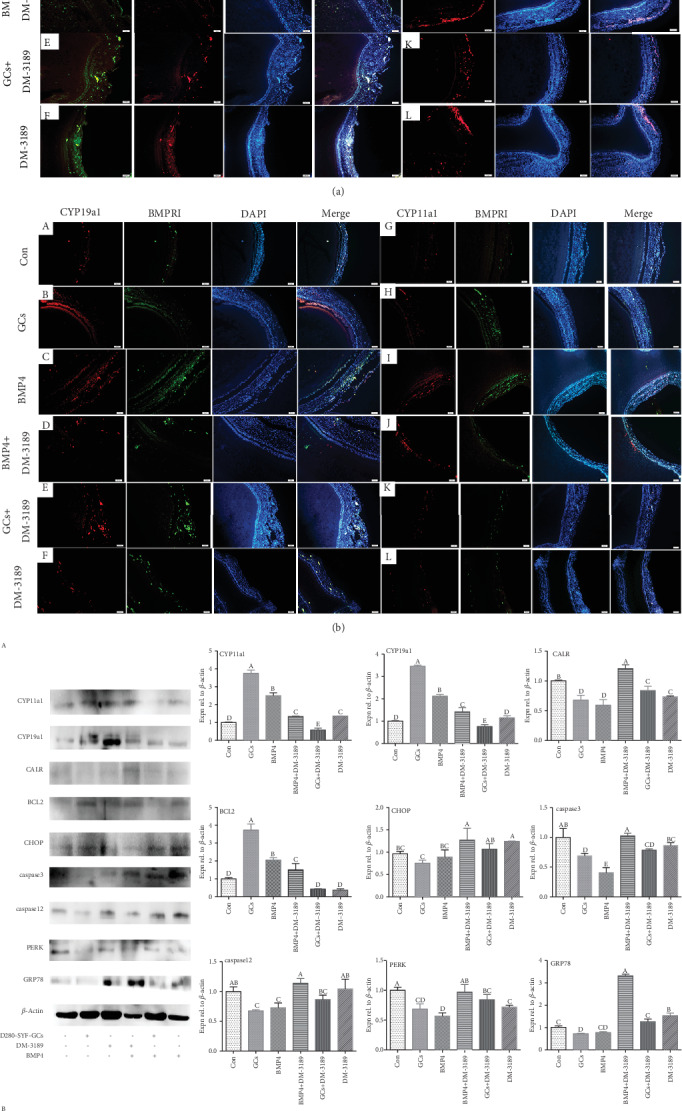
BMP4 relieved age-related ER stress and promoted steroidogenesis in SWFs. The tissue sections of the D580-SWFs were labeled with fluorescence, ER stress marker GRP78 (green), and cysteine apoptosis factor caspase3 (red), which were coexpressed ((a) A–F) in the GCs of the follicle and slightly distributed in the TCs. BrdU-positive ((a) G–L) cells (red) were distributed in the GL of D580-SWFs after treatment with BMP4. Red: CYP11a1 and CYP19a1; green: BMPR1A; CYP11a1 and BMPR1A and CYP19a1 and BMPR1 were coexpressed ((b) A–L) in the GL of D580-SWFs (treated by BMP4 or GCs). Scale bar: 50 *μ*m. Western blot and grey analysis ((c) A) of CYP11a1, CYP19a1, BCL2, CALR, CHOP, caspase3, caspase12, PERK, and GRP78. qRT-PCR analyzed *ATF4*, caspase3, and *GRP78* mRNAs ((c) B) content in D580-SWFs with different treatment methods. Values were the mean ± SEM of three experiments. Different lowercase letters indicated significant difference (*p* < 0.05).

GRP78 is one of the markers for ER stress, and caspase3 is one of the signals in the apoptosis process induced by ER stress. Results from IF staining showed that GRP78 and caspase3 were coexpressed in the GCs of D580-SWFs, and their expression was decreased after coculture with D280-SYF-GCs or treatment with BMP4. After the addition of a BMP4 receptor inhibitor (DM-3189), the expression of both GRP78 and caspase3 was increased in the GCs of D580-SWFs. The ability of BMP4 to relieve ER stress was comparable to that of D280-SYF-GCs (Figure 6(a)). On the other hand, BMP4 also promoted the expression of steroid hormone synthesis precursors (CYP11a1 and CYP19a1) in the GCs after follicle culture. The ability of the GCs to promote CYP19a1 expression was comparable to BMP4, but their effect on increasing CYP11a1 expression was inferior to BMP4 (Figure 6(b)). More importantly, the BrdU-positive cells in the GCs of D580-SWFs were significantly increased after challenge with BMP4 (Figure 6(a)). In order to reveal the relationship between ER stress and BMP4 action, ER stress-related proteins (CALR, BCL2, CHOP, caspase3, PERK, and GRP78) were detected by Western blot. Results showed that ER stress was alleviated by BMP4, and this function was equivalent to GCs from D280-SYFs. Simultaneously, the relieving function of BMP4 on ER stress was diminished after blockade of BMP receptor I (BMPRI) with DM-3189 (Figure 6(c) A). The qRT-PCR analysis confirmed these results (Figure 6(c) B).

### 3.9. Role of CALR Involved in the Effect of BMP4 on ER Stress

**Figure 7:**
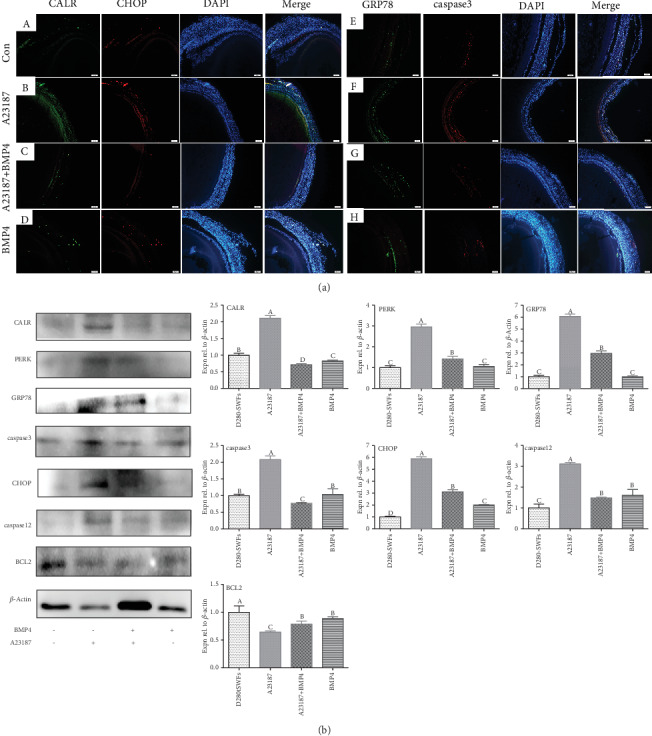
BMP4 relieved ER stress through CALR in chicken SWFs. The sections of the SWFs were immunofluorescent labeled ((a) A–D), and the ER stress marker CALR (green) and the ER stress apoptosis transcript CHOP (red) were mainly distributed in the GL, and a small amount was distributed in the TL. Histological sections of SWFs were given immunofluorescent labels ((a) E–H) with ER stress marker GRP78 (green) and apoptosis marker caspase3 (red), and they were coexpressed in the GL. Scale bar: 50 *μ*m. Western blot and grey analysis (b) of CALR, PERK, GRP78, caspase3, CHOP, caspase12, and BCL2. Values were the mean ± SEM of three experiments. Different lowercase letters indicated significant difference (*p* < 0.05).

After treatment with A23187 to activate CALR that was a marker of ER stress, both CALR and CHOP were coexpressed in the GCs of D280-SWFs by IF staining. However, their expression was reduced after treatment with BMP4 (Figure 7(a) A–D). After activation of CALR, the expression of GRP78 and caspase3 was elevated in the GCs of D280-SWFs (Figure 7(a) E–H). Furthermore, the expression of ER stress-related proteins (CALR, PERK, CHOP, caspase12, and caspase3) and the antiapoptotic protein BCL2 were detected after CALR activation, and the Western blot analysis showed that these proteins were upregulated except BCL2. Treatment of BMP4 restored all these changes (Figure 7(b)).

## 4. Discussion

The ovary is the organ that shows the earliest sign of aging in females. Follicles are gradually exhausted as females grow older, and changes in ovarian microenvironment lead to poor quality of the remaining follicles [27]. In this study, we collected ovaries from the D280 and D580 hens and compared the follicle arrangement from large to small according to their diameter. We found that the number of PHFs in D580 hens was significantly reduced as compared with that in D280 hens, which was consistent with a previous study [28]. IF staining showed that FSHR was located in GCs of the SWFs, which was consistent with a previous report [1]. Furthermore, the expression of FSHR in PHFs of D280 hens was higher than that of D580 hens. In addition, results of Western blot analysis revealed that the expression of apoptosis-related proteins (caspase3 and Bax) increased in the aging hens. These results indicated that the number of PHFs and their predominance were decreased in the old D580 hens.

Persistent ER stress response was a vital cause of cell apoptosis [29, 30]. TEM observation and IF staining demonstrated that ER stress appeared in whole follicles, whereas ER stress mainly occurred in the GCs of follicles in D580 hens, accompanied with ER degenerative changes (swelling, hyperplasia, concentric circles, etc.) [12]. Subsequently, we examined the expression of GRP78 and CALR (ER stress markers), the key transcription factor ATF4 in the downstream of the UPR signaling pathway, and the ER stress-mediated apoptotic transcription factors CHOP and caspase12 in the follicles. The results showed that the expression of these transcription factors was higher in the PHFs of the D580 hens than D280 hens, which was consistent with the previous study in humans [31].

The survival of GCs was very important for the growth and development of ovarian follicles [8]. Therefore, we isolated the GCs from the SYFs and cocultured D580-SYF-GCs with D280-SWFs or cocultured D280-SYF-GCs with D580-SWFs. Results of HE staining and TUNEL assay displayed that both the GCs and TCs of the D280-SWFs cocultured with D580-SYF-GCs manifested increased apoptosis, and the apoptotic cells were mainly distributed in the GCs. However, the apoptosis of D580-SWFs cocultured with D280-SYF-GCs was greatly reduced. On the other hand, results of IF staining showed that D580-SYF-GCs induced ER stress in D280-SWFs in coculture. Furthermore, GRP78 was mainly located in the GCs of D280-SWFs, with lower expression in the TCs. Importantly, D280-SYF-GCs alleviated the apoptosis of D580-SWF-GCs that was caused by continuous ER stress due to aging. BrdU incorporation showed that D280-SYF-GCs promoted cell proliferation in D580-SWFs. These results indicated that GCs were the first cell type that undergoes apoptosis at the initial stage of follicular dominance decline. This result is in accordance with the report by Shahrestanaki et al. [32]. Results from Western blot verified that CALR, GRP78, and key transcription factors in the pathways of the ER stress (CALR, p-PERK, PERK, ATF4, ASK1, caspase12, and CHOP) were decreased in D580-SWFs after coculture with D280-SYF-GCs.

Our study showed that apoptosis induced by ER stress is mainly through two pathways. Firstly, continuous unfolded protein reaction or Ca^2+^ imbalance induced ER stress. After that, GRP78 dissociated from three transmembrane mediators and induced apoptosis through the PERK-CHOP-BCL2/caspase3 pathway that was consistent with previous reports [16, 33]. Secondly, overactivation of calcium reticulum protein disrupted the balance of Ca^2+^ and reduced BCL2 expression leading to activated caspase12 and apoptosis. The specific mechanism of CALR-mediated apoptosis is not clear and needs further investigation [34, 35].

GCs are capable of secreting various factors to promote follicular growth and development [6–8]. Our study showed that D280-SYF-GCs could alleviate ER stress of D580-SWFs and promote follicular cell proliferation. We assumed that some cytokines secreted by the GCs are involved in this action. Based on the RNA-seq results, we found that the expression patterns of the BMP family were significantly different between the D280 and D580 PHFs. The expression of BMP family-related genes including *BMP* receptor, *TGFB*, and *TGFBR3* in D280 PHFs was higher than that in D580 PHFs. More importantly, the expression pattern of steroid hormone synthesis precursor-associated genes (*CYP11a1*, *CYP17a1*, *CYP19a1*, and *STAR*) was also significantly different in these two-type follicles. Meanwhile, BMP regulated the synthesis and secretion of steroid hormones in the follicles. On the other hand, previous studies reported that BMP4 supported the initial differentiation of chicken GCs, promoted the survival of GCs, and increased the diameter of oocytes in the primordial and secondary follicles [17, 18]. Therefore, BMP4 was selected as the main intraovarian cytokine to explore whether it has the function to alleviate ER stress of D580-SWFs.

Determination of the cytokine BMP4 demonstrated that the level of this cytokine was increased in the coculture medium of D280-SYF-GCs as compared with D280-SYF-GCs plus D580-SWFs or D580-SWFs. BMP4 promoted the expression of steroidogenic enzymes (CYP11a1, CYP19a1) and improved the survival of GCs as reported by Liu et al. [19]. Results of IHC also verified that D280-SYF-GCs increased STAR protein in GCs of D580-SWFs. More importantly, GRP78 and caspase3 were coexpressed in D580-SWFs, and this coexpression disappeared after treatment with BMP4. The expression of CALR, GRP78, PERK, CHOP, caspase12, and caspase3 proteins in D580-SWFs was all decreased while BCL2 was increased after BMP4 challenge. However, it is not clear how BMP4 regulates ER stress. De Almeida et al. found an antagonistic relationship between CALR and BMP4 [23], and the action of CALR served as a marker of ER stress [36]. Our results also showed that both ER stress and Ca^2+^ imbalance-induced apoptosis were closely related to CALR status. Therefore, we administered exogenous A23187 in D280-SWFs to induce CALR activation and then evaluated the effect of BMP4 on alleviating the sustained stress of ER that was caused by CALR. The result showed that the expression of CALR, GRP78, PERK, CHOP, caspase12, and caspase3 was downregulated after treatment with BMP4 while coexpression of GRP78 and caspase3 was increased in the GCs of D280-SWFs after the addition of BMP4 and A23187. Meanwhile, the coexpression of CALR and CHOP in the GCs of D280-SWFs was also reduced. However, the mechanism of BMP4 to antagonize CALR still remains unclear.

Several cytokines are reported to be associated with ER stress. For example, GC-secreted PGE_2_ can promote expression of GRP78, CHOP, phosphor-IRE1, cleaved ATF6, and phosphor-eIF2S1 [24, 36]. Furthermore, X-Box binding protein 1 (XBP1) is a kind of basic leucine zipper (bZIP) protein, a member of the family of transcription factors, and is activated by IRE1 shear in the ER stress. After interference of XBP1, CHOP was cleaved, followed with reduced secretion of estrogen from GCs. Meanwhile, the expression of caspase3 was increased to inhibit BCL2 expression, and it induced cell death [37]. These factors provide valuable information for measurements to alleviate age-related apoptosis in ovarian follicles.

## 5. Conclusions

**Figure 8:**
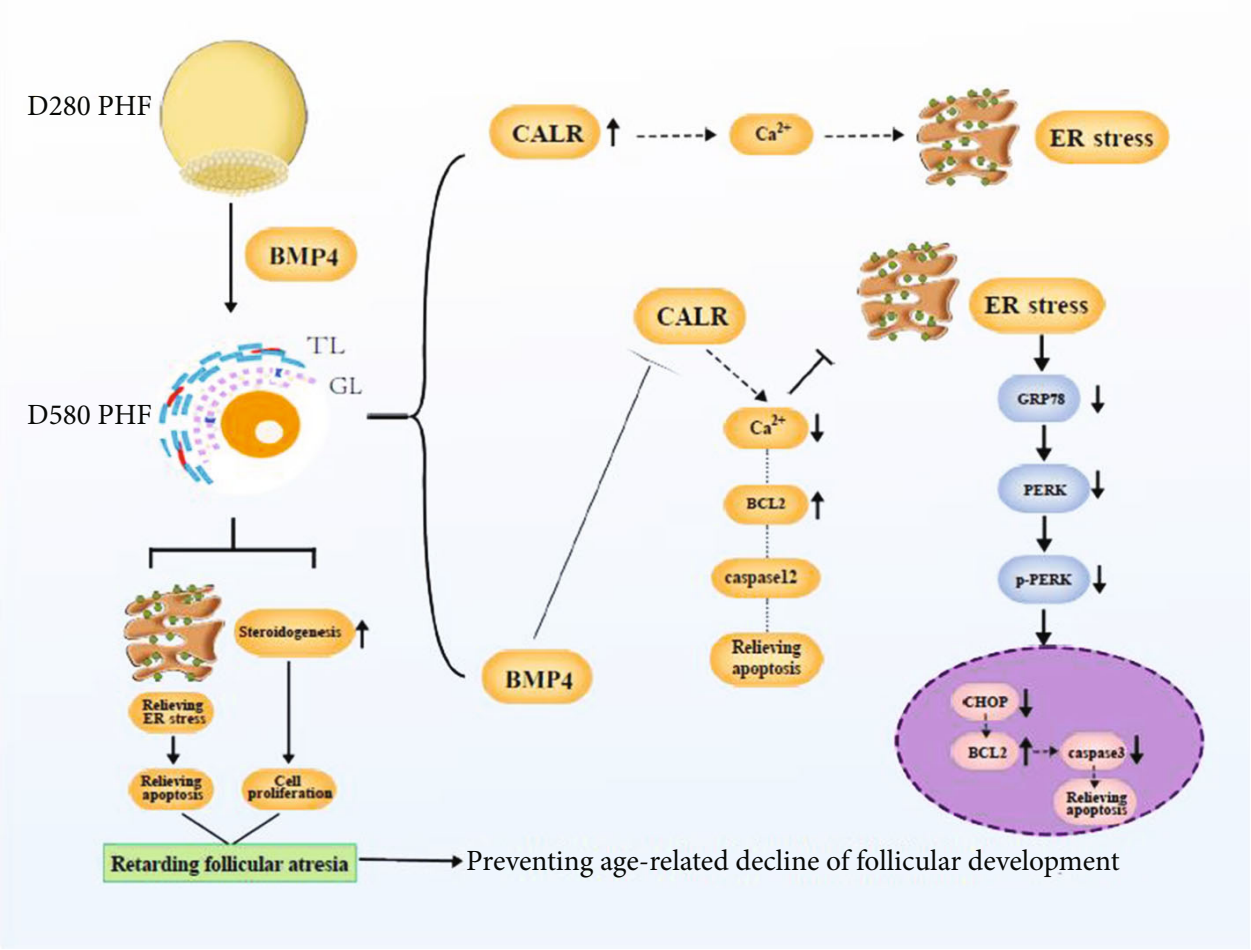
Action of BMP4 in alleviating age-related ER stress in the aging chicken.

In summary, this study proved that apoptosis of GCs was a major reason involved in the decline of dominance and delayed development in ovarian follicles, and persistent ER stress caused apoptosis in GCs of the aging chickens. GC-secreted BMP4 antagonized CALR to alleviate age-related ER stress by inhibiting the CALR-GRP78-PERK-CHOP-BCL2/caspase3 or CALR-Ca^2+^-BCL2-caspase12 pathway (Figure 8). Therefore, intensified ER stress and apoptosis occurred in GCs of the PHFs in the aged hens, while intraovarian BMP4 could improve follicular viability by alleviating ER stress and promoting follicular development.

## Figures and Tables

**Table 1:** Sequences of the primers for qRT-PCR.

Gene name	Accession number	Primer sequence (5′-3′)	Product length (bp)
*GRP78*	NM_205491.1	GAATCGGCTAACACCAGAGGA	118
		CGCATAGCTCTCCAGCTCATT	
*ATF4*	AB013138.1	TGAGCCTCTTGAACAACGAG	298
		TGTTCCATACCTAACAGGGC	
*CHOP*	XM_015273173.2	GGCCTGGTTCAATATGGGGA	117
		AATGTCTGCATAGGACACTGGT	
*PERK*	NM_001275354.1	TTTTTCAAGGCACCGCACAG	74
		GTTGGCAGCGTTCATGCCC	
*BMP4*	NM_205237.3	TCCGCTTCGTCTTCAACCTC	129
		TGTTTATCCGGTGGAAGCCC	
*FSHR*	NM_205079.1	ACCTGCCTGGATGAGCTAAA	136
		ATCCATGACTTGGCAGGAAG	
caspase3	NM_204725.1	CAGCTGAAGGCTCCTGGTTT	98
		GCCACTCTGCGATTTACACG	
*CALR*	NM_205128.1	GTGGAGACCCCGACAGATTG	99
		GTGGAGACCCCGACAGATTG	
Occludin	XM_025145796.1	TGTGTAAGGCCCACACCTCT	189
		AATGCCTTCCCAAAAAGCC	
*β*-Actin	NM_205518	ACACCCACACCCCTGTGATGAA	136
		TGCTGCTGACACCTTCACCATTC	

**Table 2:** Differentially expressed genes involved in TGF-*β*, proliferation, steroidogenesis, and apoptosis of chicken prehierarchical follicles (D580 vs. D280).

Gene ID	log_2_FC	Gene symbol	Description
Upregulated genes			
ENSGALG00000013510	0.34411	UTP15	U3 small nucleolar RNA-associated protein 15
ENSGALG00000014355	0.30701	STIM2	Sterile alpha motif
ENSGALG00000012077	0.44187	EDARADD	Death-like domain
ENSGALG00000015346	0.51732	NFKBIZ	Ankyrin repeat-containing domain
ENSGALG00000015346	0.027157	PARP9	Poly (ADP-ribose) polymerase
ENSGALG00000016897	0.23546	GPR180	Transmembrane receptor
ENSGALG00000016951	0.30953	MTRF1	Peptide chain release factor
ENSGALG00000017186	0.13575	BIRC2	Death-like domain
ENSGALG00000003064	0.20476	TADA1L	Histone fold
ENSGALG00000003914	0.064777	CALR3	Calreticulin/calnexin
Downregulated genes			
ENSGALG00000010346	-0.75224	TGFB3	Cystine-knot cytokine
ENSGALG00000006038	-0.28909	TGFBR3	Zona pellucida domain
ENSGALG00000006319	-0.48737	TGFBI	FAS1 domain
ENSGALG00000003242	-0.40236	STAR	Steroidogenic acute regulatory protein-like
ENSGALG00000027798	-0.21814	CDK2	Serine-threonine
ENSGALG00000001417	-0.44788	CYP11A1	Cytochrome P450
ENSGALG00000008459	-0.245	BMPR-II	Tyrosine-protein kinase
ENSGALG00000012216	-0.41054	BMPR1B	TGF-beta receptor
ENSGALG00000009365	-0.25099	CYP51A1	Cytochrome P450
ENSGALG00000025822	-0.36166	CYP1B1	Cytochrome P450
ENSGALG00000008121	-0.36436	CYP17A1	Cytochrome P450
ENSGALG00000013294	-0.49038	CYP19A1	Cytochrome P450
ENSGALG00000018639	-0.60478	Smad7a	MAD homology, MH1
ENSGALG00000007870	-0.3025	SMAD3	SMAD/FHA domain
ENSGALG00000000169	-0.211	PCNA	Proliferating cell nuclear antigen

## Data Availability

The dataset generated during this study is available from the corresponding author upon reasonable request.

## Supplementary Materials

Supplementary Materials Table S1: composition and nutrient level of the basal diets.
