# Supplementary material for: The Attenuating Effect of the Intraovarian Bone Morphogenetic Protein 4 on Age-Related Endoplasmic Reticulum Stress in Chicken Follicular Cells
Source: Oxid Med Cell Longev. 2020 Jun 8;2020:4175613. doi: 10.1155/2020/4175613 (PMC7301252; doi:10.1155/2020/4175613)
Supplement: Supplementary Materials — Table S1: composition and nutrient level of the basal diets. [file 4175613.f1.pdf]

Supplementary TABLE 1 Composition and nutrient level of the basal diets

Ingredients	Content (%)
Corn	65.5
Bran	2.5
Soybean meal	29
Premix [1]	3

[1] Supplied per kilogram of premix: dl-a-tocopherol acetate ≥ 280 mg; Sodium menadiones bisulfite, 30~96 mg; thiamine nitrate ≥ 38 mg; vitamin B2 ≥ 60 mg; pyridoxine hydrochloride ≥ 60 mg; vitamin B12 ≥ 0.2 mg; nicotinamide ≥ 420 mg; D-calcium pantothenate ≥ 420 mg; folic acid ≥ 12.0 mg; D-biotin ≥ 3.0 mg; choline chiorlde ≥ 5.6 g; vitamin A acetate, 160000~200000 IU; vitamin D3, 4.4~100 mg; Cu, 140~420 mg; Fe, 1.6×103~1.3 ×104; Mn, 1.2×103~3.0×103; Zn, 1.2×103~2.4×103; Se, 2.0~6.0 mg; I, 6.0~18 mg; methionine, 2.6%~5.2%; P, 1.8%~5.0%; Ca, 5.0%~20%; NaCl, 4.0%~9.0%; H_2O ≤ 10%.
